# Development of a Novel X-ray Compatible 3D-Printed Bone Model to Characterize Different K-Wire Fixation Methods in Support of the Treatment of Pediatric Radius Fractures

**DOI:** 10.3390/polym13234179

**Published:** 2021-11-29

**Authors:** Anna Gabriella Lamberti, Zoltan Ujfalusi, Roland Told, Dániel Hanna, Gergő Józsa, Péter Maróti

**Affiliations:** 1 Medical Centre, Department of Paediatrics, Division of Paediatric Surgery, Traumatology, Urology, and Paediatric Otolaryngology, UP Clinical Centre, 7 Jozsef Attila Str., HU-7623 Pecs, Hungary; lambi.anna.gabi@gmail.com; 2 Department of Thermophysiology, Institute for Translational Medicine, Medical School, University of Pecs, 12 Szigeti Str., HU-7624 Pecs, Hungary; 3 Department of Biophysics, Medical School, University of Pecs, 12 Szigeti Str., HU-7624 Pecs, Hungary; zoltan.ujfalusi@aok.pte.hu; 4 3D Printing and Visualization Center, University of Pecs, 2 Boszorkany Str., HU-7624 Pecs, Hungary; told.roland@pte.hu (R.T.); peter.maroti@aok.pte.hu (P.M.); 5 Department of Biochemistry and Medical Chemistry, Medical School, University of Pecs, 12 Szigeti Str., HU-7624 Pecs, Hungary; drainor2@gmail.com; 6 Research Group of Regenerative Science, Sport and Medicine, Szentagothai Research Centre, University of Pecs, 20 Ifjusag Str., HU-7624 Pecs, Hungary

**Keywords:** distal forearm fractures, biomechanics, K-wire, pediatrics, radius fracture, 3D printing, mechanical characterization, PLA, polyurethane

## Abstract

Additive manufacturing technologies are essential in biomedical modeling and prototyping. Polymer-based bone models are widely used in simulating surgical interventions and procedures. Distal forearm fractures are the most common pediatric fractures, in which the Kirschner wire fixation is the most widely used operative method. However, there is still lingering controversy throughout the published literature regarding the number of wires and sites of insertion. This study aims to critically compare the biomechanical stability of different K-wire fixation techniques. Different osteosyntheses were reconstructed on 189 novel standardized bone models, which were created using 3D printing and molding techniques, using PLA and polyurethane materials, and it has been characterized in terms of mechanical behavior and structure. X-ray imaging has also been performed. The validation of the model was successful: the relative standard deviations (RSD = 100 × SD × mean^−1^, where RSD is relative standard deviation, SD is the standard deviation) of the mechanical parameters varied between 1.1% (10° torsion; 6.52 Nm ± 0.07 Nm) and 5.3% (5° torsion; 4.33 Nm ± 0.23 Nm). The simulated fractures were fixed using two K-wires inserted from radial and dorsal directions (crossed wire fixation) or both from the radial direction, in parallel (parallel wire fixation). Single-wire fixations with shifted exit points were also included. Additionally, three-point bending tests with dorsal and radial load and torsion tests were performed. We measured the maximum force required for a 5 mm displacement of the probe under dorsal and radial loads (means for crossed wire fixation: 249.5 N and 355.9 N; parallel wire fixation: 246.4 N and 308.3 N; single wire fixation: 115.9 N and 166.5 N). We also measured the torque required for 5° and 10° torsion (which varied between 0.15 Nm for 5° and 0.36 Nm for 10° torsion). The crossed wire fixation provided the most stability during the three-point bending tests. Against torsion, both the crossed and parallel wire fixation were superior to the single-wire fixations. The 3D printed model is found to be a reliable, cost-effective tool that can be used to characterize the different fixation methods, and it can be used in further pre-clinical investigations.

## 1. Introduction

Forearm fractures are the most common fractures among the pediatric population, accounting for more than 40% of all childhood fractures [1,2]. The distal radius is the predominant location of these fractures, constituting 80% of all pediatric forearm fractures [3]. Fracture management aims to ensure sufficient reduction and stability. Primarily, these injuries are conservatively managed by closed reduction and cast immobilization [4,5]. In the case of unstable fractures, or when conservative treatment fails to achieve adequate reduction, surgical intervention is necessary. Wendling-Keim et al. stated in cases with repeated reduction maneuvers, re-displacement is avoidable through primary percutaneous pinning [6].

The gold standard operative method regarding these fractures is Kirschner wire fixation [7,8], since it is minimally invasive, quick, and easy to perform. Satish et al. achieved a mean procedure time for inserting a K-wire in seven minutes [9]. This technique does not require special instruments and is cheap. The surgical method is simple. After restoring the bone’s anatomical alignment, also known as the reduction of the bone, the K-wires are inserted distally from the fracture line and drilled into the proximal part of the fractured bone making it as stable as possible. That is the main purpose, to achieve stability with the fixation method. Many different techniques are described, but no clinical suggestion is based on a 3D printed fracture model. Although the many variations of percutaneous pinning are simple and effective methods regarding the treatment of unstable distal radial fractures [10], the different techniques have distinct complications and disadvantages. All K-wire techniques require additional immobilization in contrast to the ESIN technique [11]. Techniques utilizing embedded wires possess greatly reduced infection rates compared with those left on the surface, as shown by Hargreaves et al. [12]. On the other hand, Subramanian et al. observed no significant difference in infection rates between embedded and surface-planted wires, and recommend the latter to avoid reoperations [13].

The pediatric community has yet to establish a procedural guideline regarding the specific parameters of K-wire fixation [14,15]. Approaches used by pediatric surgeons vary regarding the number of K-wires applied and the site of insertion based on the personal preference and experience of the surgeon. To establish a guideline regarding the surgical treatment of these fractures, we must be able to objectively compare different methods. We recognize the characteristics, including the heterogeneity of patients, the disparate fractures, and the difference between the surgeons’ skill level, all of which make it nearly impossible to conduct standardized and reproducible research.

Three-dimensional (3D) printing technologies are effective tools in many fields of medicine, offering tailorable, scalable, and cost-effective solutions solving clinical challenges [16,17]. Recent studies highlighted the possibility of using fused filament fabrication (FFF) in orthotic and prosthetic device development or total hip replacement [18,19,20]. With respect to metal additive manufacturing technologies, patient-specific fracture fixation methods have been revealed [21], with expected beneficial clinical outcomes. From both the clinical and medical training aspects, temporal bone models are frequently used [22,23,24]. Moreover, 3D printing technology offers the possibility of performing tests without human participants, which are easily standardized and versatile. Nowadays, it can be considered a widely available tool in day-to-day clinical practice, offering user-friendly features and relatively low operation costs. Surprisingly, despite the advantages of additive manufacturing, there are no previous scientific results available in terms of standardized radius bone or other extremity models optimized for pediatric cases.

PLA is widely used in biomedical additive manufacturing. It is a cost-effective, biocompatible, and biodegradable material, which can be 3D printed with both industrial and desktop 3D printers [25,26]. Changing 3D printing parameters ensures the fabrication of models with different mechanical and structural properties; for example, printing orientation or nozzle speed can influence the elastic stiffness of the 3D printed object [27]. In biomedical simulations, PLA is mainly used for preoperative planning, temporal bone simulation, or patient education [28,29,30]. It is important to mention that regardless of its excellent biocompatibility, it cannot be directly used as a material to create orthopedic implantable devices such as hip, knee, or elbow implants. Despite the importance of the simulation of bones in the extremities, the literature is strongly limited in this field. Our previous findings have revealed that PLA has favorable mechanical characteristics: it can have relatively high resistance against flexural fatigue [31]; therefore, it could be used in the simulation of upper extremity bones in order to critically evaluate different surgical interventions.

To compare different fixation methods using a standardized protocol, we developed a 3D printed synthetic bone model to compare static biomechanical properties regarding three different types of K-wire fixation methods [32]. Additionally, we aimed to develop a method that can be easily reproduced, and the models can be shared with other experts. Previous research work in this field revealed custom-made, patient-specific surgical guides and models for distal forearm fractures [33,34], but they are mainly used for pre-operative planning, not for standardized evaluation of surgical interventions. Interestingly, these cases do not cover pediatric indications. The goal of this study is to reveal a standardized, cost-effective bone model, which can be produced with widely available fabrication processes and materials, such as PLA-based FFF 3D printing. Additionally, it is essential to design a method that is X-ray compatible. This feature provides surgeons with the possibility to critically evaluate the effectiveness of the procedure since the X-ray and CT data can be further processed and analyzed; for example, finite element simulations can be performed on segmented 3D models [35]. Additionally, the application of X-ray is indispensable for such interventions as K-wire fixation, and thus, the model has to be X-ray compatible as well. That means 3D printed models have a certain attenuation for X-ray, which is high enough for good visualization but also relatively low enough to be able to demonstrate the increased attenuations of overlapping layers on a measurable scale. If these requirements are surpassed, then we have the ultimate tool on our hands for the demonstration and practicing of several types of medical techniques and interventions. With the findings of the proposed method, a novel 3D printed model has been introduced, which can be used for surgical simulations and pre-clinical investigations regarding K-wire fixations in a cost-effective and standardized way. Mechanical tests and medical imaging techniques have underlined the practical usability of the simulator, which was critically evaluated by performing different K-wire fixation methods.

## 2. Materials and Methods

The design of the models and the parameters of the experiment were the results of a collaborative effort among both medical and engineering professionals. All engineering procedures were fine-tuned according to the feedback provided by a pediatric surgeon and traumatologist with twelve years of experience, thus ensuring the most optimal experimental design.

### 2.1. Basic Model Description

**Figure 1:**
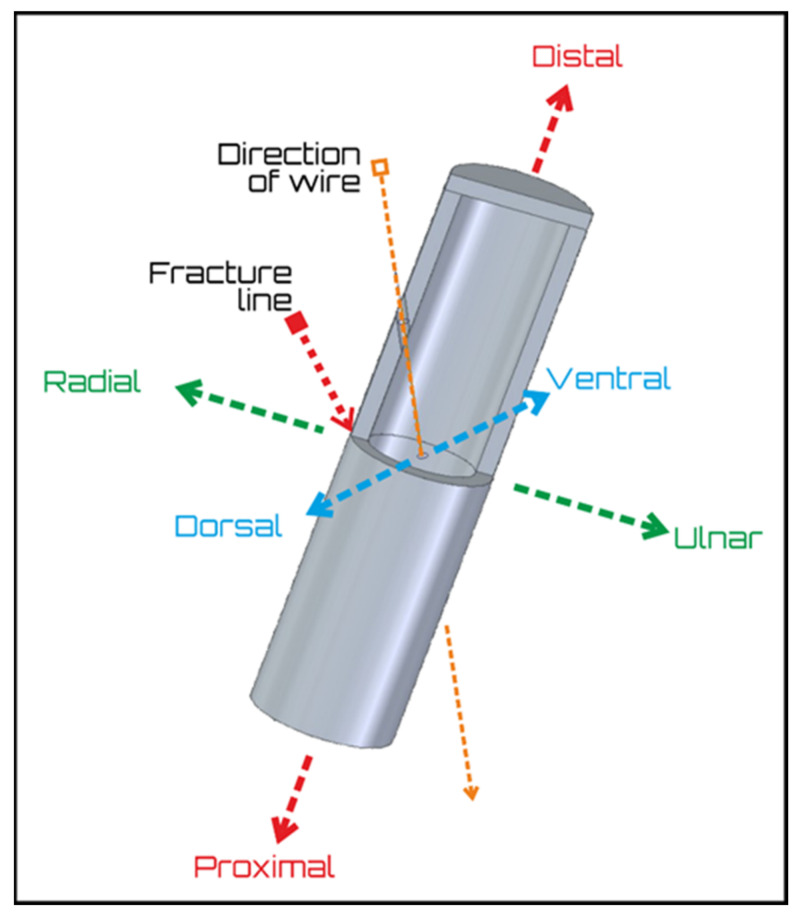
The fitted base on the bone model. The arrows define the anatomical directions, which were established in full accordance to medical terminology, including the direction of the wire and the fracture line.

The bone model was designed as a 3D printed plastic shell (the outer, cortical part of bone model) and was filled with a thermoplastic polyurethane sponge, which was intended to simulate the soft, sponge-like consistency of the internal (medullar) part of the bone. The geometry of the radius served as a base for the model. Therefore, the cross-section is an elliptic shape with the following parameters: the major axis was 25 mm, the minor axis was 18 mm, and the outer, cortical part was printed with 3 mm. The length of the ellipse-based cylinder measured 50 mm, with one side closed to prevent the foam from seeping out during pouring. To position the wire in place, the bones were designed with preformed holes. The pre-printed holes ensure the direction and location of a wire within a model and are standard features in all models. Two manufactured pieces were fastened together with their open ends to form and replicate the complete broken radius. The directions were established according to medical terminology and the specific orientation of all the wires was suitably identified (Figure 1).

**Figure 2:**
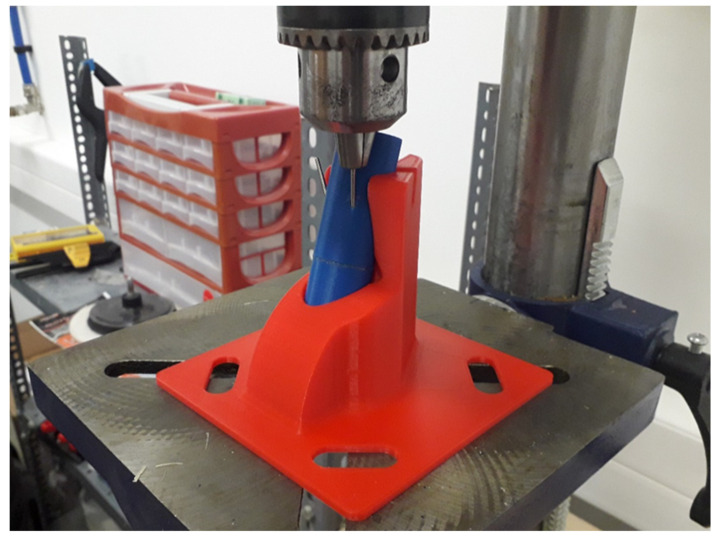
The insertion of the K-wires was mechanized by an Einhell BT-BD 701 stand drill.

For the development, commercially available materials were used. The material of the outer, cortical part of the model was polylactic acid (PLA) and Filanora Filacorn PLA BIO (Friend Plastic Ltd., HU-7300 Komló, Hungary). The filament diameter was 1.75 mm. The model was fabricated using a CraftBot XL desktop 3D printer (CraftBot XL, CraftBot Ltd., HU-1087 Budapest, Hungary). The PLA was printed with a 210 °C hot end on a 60 °C preheated tray, the nozzle diameter was 0.4 mm, and the printing speed was set to 60 mm/min with 50% infill and Z printing orientation. The printing parameters were set by the technical recommendations of the manufacturer. In addition, previous studies were investigated in order to use the optimal settings, along with the previous works of our research groups [36,37,38,39]. Regarding 3D model slicing, which prepared the printable .stl file format, the company’s default CraftWare software was used. The interior, medullary part of the model was filled using polyurethane foam Poly ON 013/80HM polyol + ISO 30 isocyanate in 1:1 by volume (Alvin Kereskedőház ZRt., HU-2092 Budakeszi, Hungary), in which the free expansion density was 80 kg/m^3^ ± 18 kg/m^3^. With regard to each type of model, a template was 3D printed to maintain stability and the right angle regarding proper fixation. The K-wire was inserted in the chuck of an Einhell BT-BD 701 stand drill (Einhell BT-BD 701, iSC GmbH, D-94405 Landau an der Isar, Deutschland), and the wire was inserted into the model (Figure 2).

Additionally, 15 full-length test samples (100 mm) for the mechanical and structural characterization were fabricated in order to validate the practical usability of the proposed method. In the text, this model is referred to as an “intact model” A cost estimation has also been carried out.

The models for testing the different fixation methods (∑n = 189) were divided into three groups (Group I: n = 27, Group II: n = 27), and the third group was divided based on the specific configuration regarding the fixation method (Group III with an additional four subgroups, n = 27 for each subgroup). Each fixation method has been tested nine times with all testing methods (n = 9).

### 2.2. Groups

**Figure 3:**
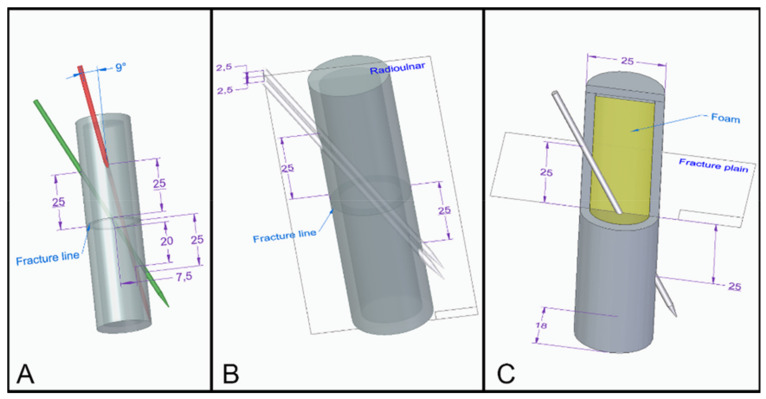
(**A**) Group I—crossed wires; (**B**) Group II—parallel wires; (**C**) Group III—single wire fixation. The purple arrows depict the location of the entry and the exit points of the wire and the diameters of the model. The blue arrows represent the angle between the wires at the crossed wires fixation. The black rectangles are characteristic of the various planes.

Group I (n = 27): the K-wire fixation was performed using two crossed wires. The entry point of the first wire was on the radioulnar plane 25 mm from the fracture line in the distal direction, and the exit point was 25 mm from the fracture line in the proximal direction. The entry point of the second wire was on the dorsoventral plane 25 mm from the fracture line in the distal direction, and the exit point was 20 mm from the fracture line in the proximal direction and 7.5 mm from the dorsoventral plane in the ulnar direction (at a nine-degree angle) (Figure 3A).

Group II (n = 27): the K-wire fixation was performed using two wires parallel with the radioulnar plane ± 2.5 mm in the dorsal and ventral directions, with entry points 25 mm from the fracture line in the distal direction and exit points 25 mm in the proximal direction (Figure 3B).

**Figure 4:**
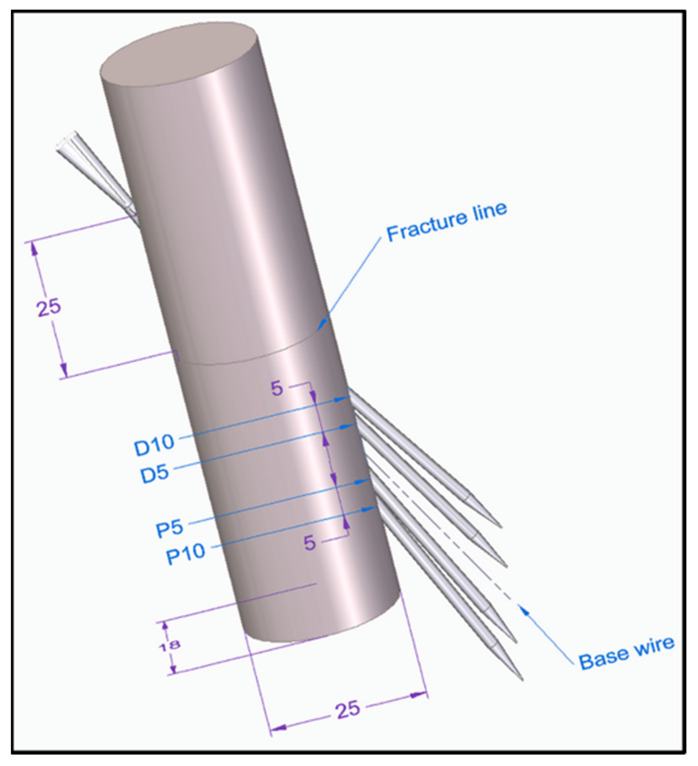
The dashed line represents Group III (base wire). In the figure, four different fixation setups (gray needles) are illustrated on a single model to visualize the difference between the Group III. Subgroups: D10—distal 10 mm, D5—distal 5 mm, P5—proximal 5 mm, P10—proximal 10 mm. The purple arrows depict both the location of the entry and the exit points of the wire including the diameters of the model.

Group III (n = 27): the K-wire fixation was performed using one wire, which was inserted on the radioulnar plane 25 mm from the fracture line in the distal direction with an exit point 25 mm in the proximal direction from the fracture line (Figure 3C). This design served as the basis of the Group III subgroups (Figure 4).

Group III/D10 (n = 27): the K-wire fixation was performed using one wire as described above, with an exit point shifted 10 mm distally (D10) when compared with Group III.

Group III/D5 (n = 27): the K-wire fixation was performed using one wire as described above, with an exit point shifted 5 mm distally (D5) when compared with Group III.

Group III/P5 (n = 27): the K-wire fixation was performed using one wire as described above, with an exit point shifted 5 mm proximally (P5) when compared with Group III.

Group III/P10 (n = 27): the K-wire fixation was performed using one wire as described above, with an exit point shifted 10 mm proximally (P10) when compared with Group III (Figure 4).

### 2.3. Mechanical and Structural Tests, Imaging

#### 2.3.1. Mechanical Testing of the Intact and Fixed Models

**Figure 5:**
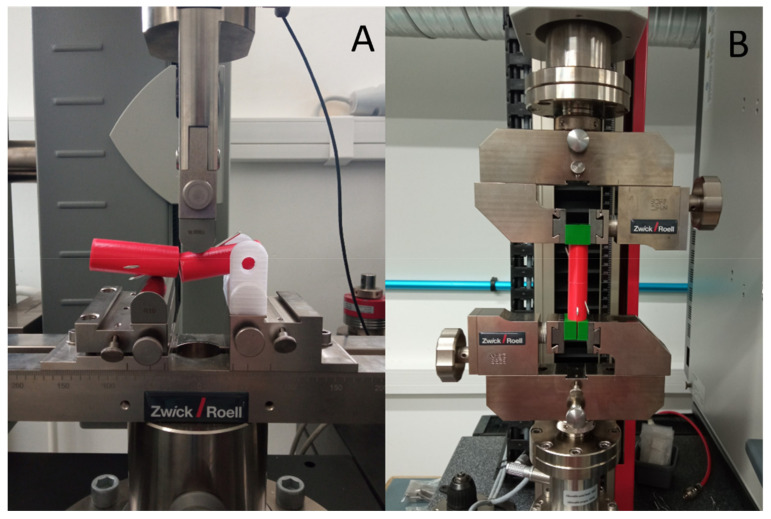
The mechanical testing setups. (**A**) Three-point bending test setup. In the picture the test is in progress, the model is under dorsal load. (**B**) Torsion test setup. The machine is in resting position, no torque is applied.

As a first step, the intact models have been measured and characterized. Five test samples have been measured, and they served as a control group. Following the initial measurements, the different fixed models have been tested within the three groups and four additional subgroups, repeating each test nine times. The same testing settings were used in both cases (Figure 5). The aim of the mechanical testing was two-fold. On one hand, it was intended to provide detailed information regarding the mechanical behavior, reliability, and usability of the bone model; on the other hand, the effectivity of K-wire fixations has been investigated and compared by these methods.

##### Three-Point Bending Test

**Figure 6:**
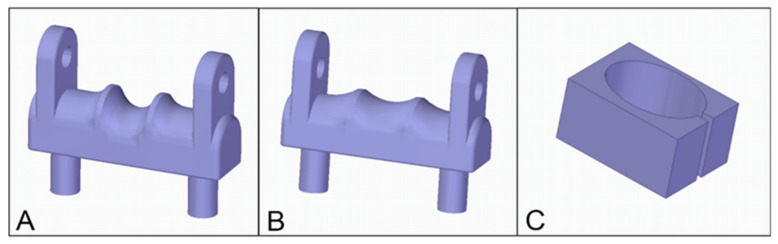
These 3D printed devices stabilized the models during the stress tests; (**A**) the template to the radial load; (**B**) the template to the dorsal load; (**C**) and the adapter to the torsion test. Reference for models can be found in the Appendix A, Appendix B, Appendix A, Appendix A, Appendix C, Appendix D, Appendix E, Appendix F and Appendix G.

The three-point bending tests were carried out using a ZwickRoell Z100THW universal material tester (ZwickRoell Z100THW, ZwickRoell, D-89079 Ulm, Germany) with a 1 kN load cell (Figure 5A). One of the supports was altered to a geometric shape, which replicated the bone model, also ensuring radial and dorsal load (Figure 6A,B; Appendix A, Appendix B, Appendix A, Appendix A, Appendix C, Appendix D, Appendix E, Appendix F and Appendix G). The pre-load was 0.05 N, the testing speed was set to 2 mm/min, and the measurement was stopped at a 5 mm distance transversed by the crosshead. The applied force did not load the model at the fracture line, but at 13 mm from it in distal direction. The support distance was 54 mm. The measurement was carried out on nine models per group and subgroup, and the maximum force values were analyzed.

##### Torsion Test

The torsion tests were carried out using ZwickRoell Z5.0 Biaxial material tester (ZwickRoell Z5.0, ZwickRoell, D-89079 Ulm, Germany) (Figure 5B). In consideration of the screw grips, an adapter was 3D printed, exactly matching the bone model (Figure 6C). The pre-torque was 0.05 Nm, the test speed was set to 60°/min, and measurement was stopped at 180° rotation. The measurement was carried out using nine models per group and subgroup. The torque values were measured at 5° and 10°, since these are clinically relevant values. Below 5° torsion, spontaneous recovery is expected; between 5° and 10°, complications are more likely to occur, and above 10°, torsion physiological remodeling is not possible.

##### Imaging with Digital Microscopy and X-ray

To gain a better insight into the mechanical behavior of the bone model and to determine its clinical usability, digital microscopy imaging, and digital X-ray imaging have been performed. The digital microscopy was carried out using a König Digital microscope, with 35× magnification. The digital X-ray sensor provided further analysis of the specimens with the possibility to instantly visualize accidental errors in the printouts and in the filling. Cracks and other damages can also be detected together with the accurate position of the applied metal wires. The grayscale shades of specimens examined under the same experimental setup can be evaluated, and thus the attenuation of certain parts of different samples can be measured and compared. For these investigations, we used Phywe XR 4.0 expert unit with an XR 4.0 direct digital image sensor (Phywe XR 4.0, PHYWE Systeme GmbH & Co. KG, D-37079 Göttingen, Germany). The detector of the instrument captures 16-bit grayscale TIFF images with a resolution of 500 × 500 pixels. The evaluation of the obtained TIFF images has been performed with Scion Image for Windows 4.0.2. The grayscale resolution of the images decreased to 8 bits after importing them in Scion Image. By applying the required built-in macro, grayscale values have been attributed to the selected area of pixels (an integer from 0 (black) to 255 (white)). On each image, we evaluated the average grayscale value of a 225 square pixel area (15 × 15 pixels). That size was suitable for us to quantify the grey shades, but, of course, that is subject to change according to the type of the sample and the preferences of the operator. The obtained greyscale values are consistent and suitable for cross-sample analysis.

#### 2.3.2. Statistical Analysis

The data acquired from the material testing devices were analyzed using Jamovi 1.8.1 software. All groups were tested for normal distribution by the Shapiro–Wilk test and Q-Q plots [40]. The homogeneity of variances was established by Levene’s test. Analysis of variances (ANOVA) was performed to determine differences between the strength of fixation methods on all groups eligible for parametric testing. In reference to the remainder of the groups, a Kruskal–Wallis test was performed as the non-parametric alternative [41]. Afterward, post hoc tests (Tukey’s test for parametric and Dwass–Steel–Critchlow–Fligner (DSCF) multiple comparisons for non-parametric analyses) were utilized to conclude the strengths and limitations of the different fixation configurations.

## 3. Results

### 3.1. Mechanical Characterization of the Intact Bone Model

**Figure 7:**
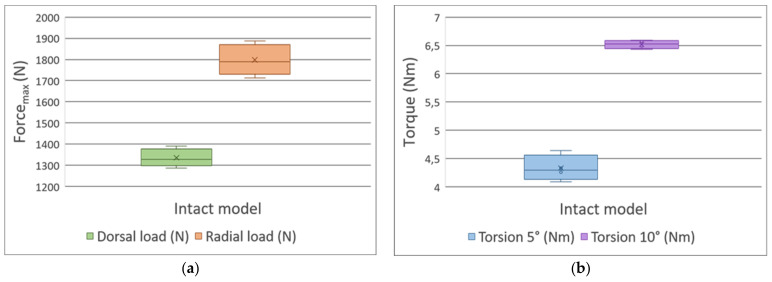
Box plots of the (**a**) three-point bending and (**b**) torsion tests regarding the intact bone models. The radial, dorsal loads, and the 5° and 10° torsions are coded using various colors.

The measurements with the intact model have revealed the mechanical characteristics and mechanical behavior of the proposed polymer-based model. In the case of the dorsal load, fmax was measured as 1335.4 N ± 41.7 N, while the radial load was 1798.2 N ± 72.3 N and the deflection was 5 mm (Figure 7a). The mean values for torsion tests resulted in 4.33 Nm ± 0.23 Nm with 5° rotation, and 6.52 Nm ± 0.07 Nm with 10° rotation (Figure 7b; Appendix A).

### 3.2. Comparison of Fixation Methods

Following the initial mechanical characterization of the intact model, the comparison of different fixation methods has been carried out based on the predefined groups.

#### 3.2.1. Three-Point Bending Test—Dorsal Load

**Figure 8:**
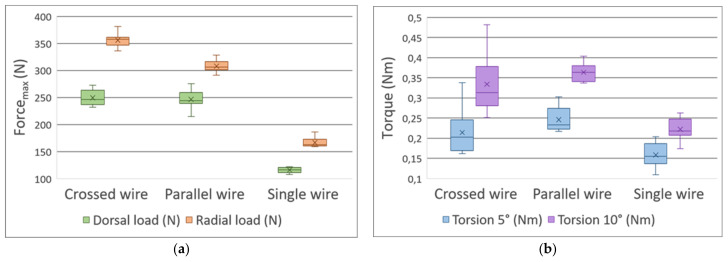
Box plots of the (**a**) three-point bending and (**b**) torsion tests, regarding the different fixation methods. The radial, dorsal loads, and the 5° and 10° torsions are coded using various colors.

There was no significant difference between Groups I and II: their values were 249.0 N ± 14.1 N (mean ± SD) and 246.0 N ± 16.9 N, respectively (mean difference = 3.1 N). The dorsal load was significantly higher in Groups I and II compared to Group III: 116.0 N ± 5.2 N (Group I vs. Group III, *p <* 0.001 and Cohen’s d = 10.3, mean difference = 133.6 N; Group II vs. Group III, *p <* 0.001 and Cohen’s d = 10.0, mean difference = 130.5 N) (Figure 8a; Appendix B, Appendix C and Appendix D).

#### 3.2.2. Three-Point Bending Test—Radial Load

There was a significant difference between Groups I and II: their values were 356.0 N ± 12.6 N and 308.0 N ± 10.8 N, respectively (*p <* 0.001 and Cohen’s d = 4.3, mean difference = 47.8 N). The radial load was significantly higher in Groups I and II compared to Group III; 166.0 N ± 9.5 N (Group I vs. Group III, *p <* 0.001 and Cohen’s d = 17.1, mean difference = 189.4 N; Group II vs. Group III, *p <* 0.001 and Cohen’s d = 12.8, mean difference = 141.6 N) (Figure 8a; Appendix B, Appendix C and Appendix D).

#### 3.2.3. Torsion Test

No differences were found in the torsion test between Groups I and II: at 5°, the values were 0.214 Nm ± 0.058 Nm and 0.246 Nm ± 0.032 Nm; at 10°, 0.334 Nm ± 0.072 Nm and 0.364 Nm ± 0.023 Nm, respectively. It is clear, however, that Group I showed far greater dispersion (as the standard deviations above indicate). There was a significant difference between Group III and Groups I and II (Group I and II: *p <* 0.05 with an ε2 > 0.6 at both 5°and 10°, suggesting a strong effect) [42] (Figure 8b; Appendix B, Appendix E and Appendix F).

### 3.3. The Distance between the Fracture Gap and the Exit Point Regarding the K-Wires Affected the Stability of the Fixation

#### 3.3.1. Three-Point Bending Test—Dorsal Load

**Figure 9:**
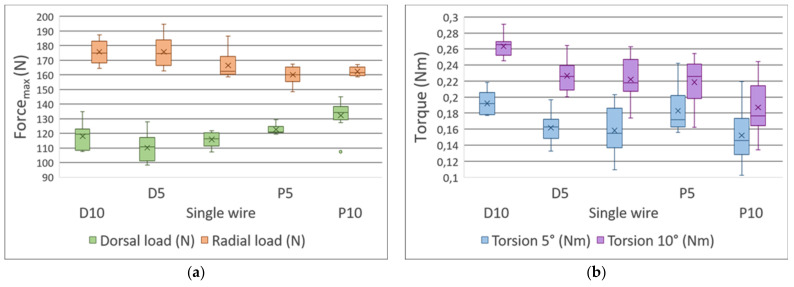
Box plots of the (**a**) three-point bending and (**b**) torsion tests representative of Group III and Group III subgroups. The radial, dorsal loads, and the 5° and 10° torsions are coded using various colors.

There was a significant difference between the maximum dorsal load between the groups with a significant effect size (*p <* 0.001, η^2^ = 0.489), and we observed that shifting the exit point proximally increases the maximum dorsal load (Group III/D10 < Group III/P10 at *p <* 0.005 and Cohen’s d = −1.762, mean difference = 14.2 N) (Figure 9a; Appendix G, Appendix C, Appendix D).

#### 3.3.2. Three-Point Bending Test—Radial Load

The maximum radial load also depicted a significant difference with a consequential effect impacting size (*p <* 0.001, η^2^ = 0.441); however, in contrast to the dorsal load, a distal shift of the exit point produced better results (Group III/D10 > Group III/P10 at *p <* 0.007 and Cohen’s d = 1.705, mean difference = 13.4 N) (Figure 9a; Appendix C, Appendix D and Appendix G).

#### 3.3.3. Torsion Tests

In general, the distally shifted exit point resulted in greater resistance to torsion in both experimental setups (at 5°, *p <* 0.01, η^2^ = 0.275; at 10°, *p <* 0.001, η^2^ = 0.498). It is noteworthy that at 5° torsion, the post hoc test revealed no statistically significant difference between the groups, except for Group III/P5 and D10 (p > 0.05 in all cases). The biggest difference was observed between Group III/D10 and Group III/P10 at 10° torsion (*p <* 0.001, Cohen’s d = 1.788, mean difference = 0.076 Nm) (Figure 9b; Appendix C, Appendix D and Appendix G).

### 3.4. Results of Imaging

**Figure 10:**
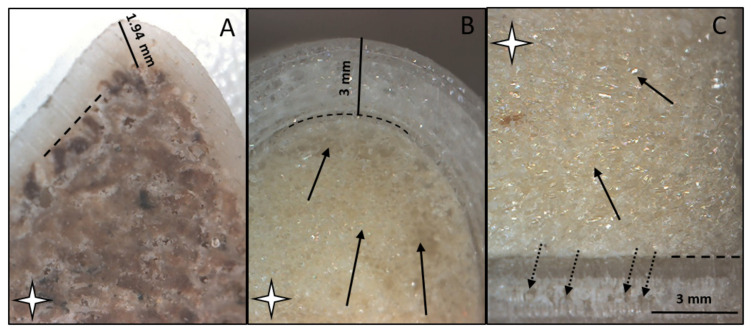
Image (**A**) demonstrates the structure of the demonstrational swine bone (cross section). Image (**B**) demonstrates the horizontal and image (**C**) demonstrates the perpendicular cross-section of the fabricated bone model. The black lines represent the outer, cortical parts, which is measured as 1.94 mm in case of the swine bone, and 3.00 mm in case of the 3D printed model. The white star indicates the medullar part of the bone and the simulated medulla of the model. The dashed black lines represent the transition between cortical and medullar parts. The black arrows indicate the porous structure of the model, made of polyurathane. Pointed black arrows point to the interlayers space, which demonstrates the 50% infill density of the cortical part. All images were made with 35x magnification.

The macroscopic structure of the bone model was compared with swine bone (distal part of femur), whose bone is not related to any animal study. The bone is sterilized, boiled, dried, and preserved for education purposes. The cuts of cross-sections have been performed with a handheld saw, and it has no effect on the performed experiments. On the microscopy images, it is clearly seen that the macroscopic structure of the developed model is similar to swine bone; the cortical and medullar parts can be visually separated. The cortical part is dense, and the transition is continuous to the spongy, foam-like structure (Figure 10).

**Figure 11:**
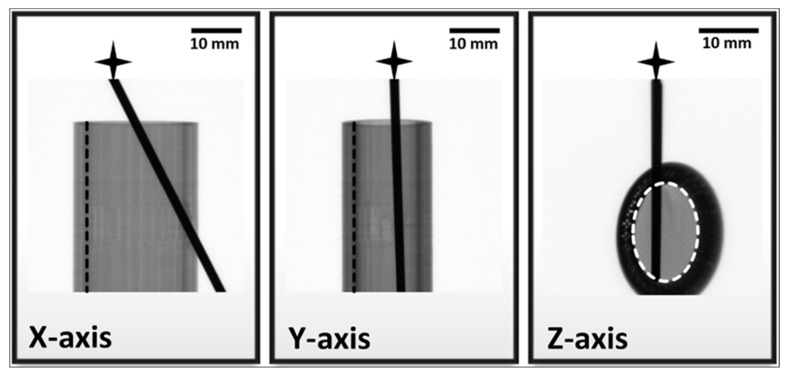
X-ray images taken from the same PLA-PU model-bone specimen in all three planes. The elevated attenuation caused by the increased sample thickness is observable with naked eye. The black star indicates the K-wire rod, and the dashed black and white lines represent the border of the simulated cortical and medullar regions.

The cortical part was set to 3 mm in the case of the model, which relies on clinical experience [43]. The porous structure visually simulates the bone marrow. CT imaging is essential in medical education and simulation; therefore, compatibility with clinical imaging devices is crucial in terms of any bone model. X-ray and computed tomography are excellent methods to visualize the accurate position and incidental deformations of metal implants in either the 3D printed specimens or, e.g., in tubular bones. Still, X-ray images were taken of the model specimen from different angles to demonstrate the benefits of this type of imaging on implant models. The visualization of the grayscale density alterations of certain components provides a higher level of evaluation and accuracy for the professionals (Figure 11).

The differences in sample thickness in the X and Y planes are represented by the evaluated 8-bit greyscale values obtained from images captured at those planes (Table 1). Table 1 also contains the greyscale values of the medical steel–alloy rod pierced through the sample in each plane, as a reference for the darkest shade on the sample. The differences in greyscale values reliably demonstrate the material- and thickness-related attenuations of the dense PLA coating and the foam-like PU filling.

## 4. Discussion

K-wire fixation is a long-established surgical intervention regarding the treatment of pediatric distal forearm and radius fractures. Surgical interventions can be evaluated in different ways. Complication rates, costs, operative time, duration of hospitalization, time interval to wire removal, functional results, residual symptoms, etc., can all serve as a basis for effective evaluation regarding operative techniques.

The primary stability assessment of fracture fixation techniques today is based on ex vivo cadaver testing. This method is expensive and limited by the number of available human tissue and therefore is not suitable for large-scale testing [44]. In terms of pediatric surgery, the heterogeneity of the features of the children’s bone—for example, its material composition, tensile, and compressive stiffness changes vastly with age and differs among the sexes [43]—makes it impossible to conduct meaningful experiments. Further complicating the situation ethical considerations weigh heavily when trials with human participants (especially children) are planned.

This study aims to provide a new perspective in the comparison of the biomechanical stability regarding the three most commonly used different K-wire fixation techniques. The main goal of this work was to select the K-wire fixation technique, which offers the greatest stability based on objective data and to propose a model, which can be easily reproduced by other research groups and provide a standard method to investigate surgical procedures performed on the bones of the upper extremity. It should be emphasized that this goal was set, due to the clinical need for objective information about the difference in fixation stability between commonly used surgical methods relative to each other.

Three-dimensional printing provides a versatile solution for modeling or prototyping in the biomedical field. The developed model, fabricated using PLA material with a desktop FFF 3D printer and a two-component polyurethane with basic molding techniques, was found practically useful in the characterization of different K-wire fixation methods. Based on the literature review, 3D printed bone models for the evaluation of surgical procedures related to the upper extremities have not been described previously. The mechanical characterization of the intact model revealed that the proposed method is reliable since the standard deviations are low; they varied between 1.09% (5° torsion; 6.52 Nm ± 0.07 Nm) and 5.28% (10° torsion; 4.33 Nm ± 0.23 Nm). Additionally, it is observed that the structure of the developed model is similar to the structure of a swine bone model, which is frequently used in medical education not only for demonstrational purposes, but for hands-on training as well. The visual appearance of the model can serve didactic purposes too since the cortical and medullar parts are easy to separate visually. Additionally, it can be mentioned that the cortical:medullar ratio can vary based on the anatomical points that are simulated. The novel method can also be used with standard clinical imaging techniques such as CT or X-Ray. In addition to standardization, cost-effectivity was a major aspect as well. It takes 4.66 h to prepare 10 full models using two FFF 3D printers (with a printing time of 4 h, polyurethane pot-time and post-processing: 40 min), and it costs EUR 19.1 (machine costs and utilities: EUR 15.6, PLA material: EUR 3.2 polyurethane material: EUR 0.3). This means that one piece of the bone model can be fabricated for under EUR 2, which can be considered as an extremely cost-effective solution compared to other models available on the market.

Our comparison regarding the various fixation methods aptly showed that using two K-wires for fixation resulted in significantly increased stability. The crossed wire fixation technique when compared with the parallel wire fixation resulted in greater resistance during the dorsal and radial load tests and exhibited no significant differences during the torsion tests. Based on our data, however, the crossed wire fixation technique appears to be less resistant to torsion forces.

Our secondary goal was to evaluate the effect of the distance between the fracture line and the exit point of the K-wires on stability.

The effect of the shift of the exit point with a single wire fixation did not depict conclusive results. Increasing the distance between the fracture line and the exit point of the K-wire suggested an increase in the maximum dorsal load and a decrease in the maximum radial load.

During the torsion tests, both at 5° and 10°, a decrease in the distance between the exit point and the fracture line resulted in greater resistance to torsion force. This effect corresponds with our hypothesis in which, by increasing the angle between the bone’s rotational axis and the K-wire results in increased resistance to torque. It should be noted, however, that torsion could be counteracted by a lengthy cast, so no rotation is possible.

## 5. Conclusions

The performed measurements and the statistical analysis underlined that 3D printing technology is an effective way to construct models for medical simulations. Using PLA and polyurethane can potentially be used for mimicking bones for critically evaluating surgical interventions since they can provide a reliable, standardized, and cost-effective method, which can be translated for other clinical simulations as well. In case of the intact bone model, the standard deviations varied between 1.09% (5° torsion; 6.52 Nm ± 0.07 Nm) and 5.28% (10° torsion; 4.33 Nm ± 0.23 Nm), which underlines the reliability of the model.

Conventional X-ray is the best diagnostic tool for the detection of bone fractures in adult and pediatric care. It is essential to visualize the internal conditions before (sometimes during) and after fixation. The verification of several types of fixations by X-ray is a mandatory requirement right after the implantation and later at the patient follow-ups. A good model perfectly simulates the original object and its main features in general. We have designed our model with this in mind, and the PLA-based model proved to be an excellent alternative for practicing fixations. It also has the advantage of being X-ray compatible, i.e., the attenuation of PLA at certain places allows us to distinguish cortical-like (numeric value of grayscale shades varied between 17 ± 2 and 105 ± 1) and medullar-like regions (numeric value of grayscale shades varied between 116 ± 9 and 133 ± 4), and all the fine details of the inserted fixation are also visible. The perfect visibility of the fixation throughout the sample is crucial because any kind of bending, fraction, twisting, or caused damage on the inside can accurately be detected even if the other end of the fixation is still inside and/or stuck. The presented PLA-PU model serves as a good and easy-to-prepare example for modeling tubular bones and, thus, it is a great and affordable alternative for physicians to design the steps of a given fixation before surgical intervention, or this model is a valuable tool for practicing such interventions.

We conclude, in reference to fractures of the distal radius and forearm among children, which require operative treatment, based on biomechanical stability, two crossed percutaneous K-wiring is the preferred procedure. Crossed wire fixation offered the same or better stability than any other fixation method tested. Based on our results, single wire fixation offered significantly less stability and was outperformed in every measured parameter (*p <* 0.05, Appendix C, Appendix D, Appendix E and Appendix F). Compared to parallel wire fixation, crossed wire fixation offered greater stability during radial load (mean difference = 47.8 N or 15.5%, Cohen’s d = 4.33—for interpretation see Appendix D) and showed no difference in terms of stability during radial load and torsion tests. In clinical practice, it is advantageous to use the crossed wire technique as it theoretically provides better resistance against the re-displacement of the fracture caused by muscle traction.

## Figures and Tables

**Table 1:** Quantified grayscale values on the standard 8-bit scale (integer from 0 (black) to 255 (white)) of different areas of the specimen in all three planes; values are mean + standard deviations obtained from five standalone measurements.

Numeric Values of Grayscale Shades			
	Cortical	Medullar	K-wire
X-axis	105 ± 1	129 ± 3	5 ± 1
Y-axis	69 ± 1	116 ± 9	5 ± 1
Z-axis	17 ± 2	133 ± 4	4 ± 1

## Data Availability

Data are contained within the article or in the Appendix section.

## Appendix A

**Table A1:** Descriptive statistics of the intact bone models. The table contains the means, the medians, the standard deviations, the variances, and the Shapiro–Wilk W and *p* values.

Descriptive Statistics				
	Dorsal Load (N)	Radial Load (N)	Torsion 5° (Nm)	Torsion 10° (Nm)
Mean	1335	1798	4.33	6.52
Median	1328	1789	4.29	6.53
Standard deviation	41.7	72.3	0.23	0.0739
Variance	1736	5228	0.0529	0.00547
Shapiro–Wilk W	0.975	0.961	0.957	0.927
Shapiro–Wilk *p*	0.908	0.818	0.762	0.574

## Appendix B

**Table A2:** Descriptive statistics of the different fixation methods (Group I, II, III). The table contains the means, the medians, the standard deviations, the variances, and the Shapiro–Wilk W and *p* values.

Descriptive Statistics					
	Fixation Method	Dorsal Load (N)	Radial Load (N)	Torsion 5° (Nm)	Torsion 10° (Nm)
Mean	Crossed wire	249	356	0.214	0.334
	Parallel wire	246	308	0.246	0.364
	Single wire	116	166	0.158	0.222
Median	Crossed wire	246	357	0.202	0.313
	Parallel wire	244	306	0.233	0.364
	Single wire	116	162	0.155	0.218
Standard deviation	Crossed wire	14.1	12.6	0.0576	0.0721
	Parallel wire	16.9	10.8	0.032	0.0225
	Single wire	5.15	9.45	0.0303	0.0272
Variance	Crossed wire	198	160	0.00332	0.0052
	Parallel wire	285	117	0.00103	5.07 × 10^−4^
	Single wire	26.5	89.2	9.21 × 10^−4^	7.37 × 10^−4^
Shapiro–Wilk W	Crossed wire	0.935	0.959	0.834	0.909
	Parallel wire	0.944	0.974	0.812	0.947
	Single wire	0.933	0.781	0.974	0.96
Shapiro–Wilk *p*	Crossed wire	0.531	0.792	0.049	0.307
	Parallel wire	0.627	0.927	0.028	0.656
	Single wire	0.509	0.012	0.927	0.796

## Appendix C

**Table A3:** Results of the ANOVA tests. The level of significance was set to 5% (α = 0.05).

ANOVA—Test Results							
		Sum of Squares	df	Mean Square	F	p	η^2^
Fixation method	Dorsal Load (N)	104,690	2	52345	309	< 0.001	0.963
	Radial Load (N)	174,647	2	87324	715	<0.001	0.983
Subgroups of single wire fixation	Dorsal Load (N)	2475	4	618.8	9.56	<0.001	0.489
	Radial Load (N)	1951	4	487.8	7.87	<0.001	0.411
	Torsion 5° (Nm)	0.0107	4	0.00268	3.8	0.01	0.275
	Torsion 10° (Nm)	0.0265	4	0.00662	9.94	<0.001	0.498

## Appendix D

**Table A4:** Results of the post hoc (Tukey) tests. The level of significance was set to 5% (α = 0.05). Statistically significant (ptukey < 0.05) results are highlighted with light gray.

Post Hoc Comparisons—Test Results							
	Comparison						
Experiment Setup	Name of the Group		Name of the Group	Mean Difference	t	**p_tukey_**	Cohen’s d *
Fixation method, dorsal load (N)	Crosswire	-	Parallel Wire	3.13	0.51	0.867	0.24
		-	Single Wire	133.63	21.765	<0.001	10.26
	Parallel Wire	-	Single Wire	130.5	21.255	<0.001	10.02
Fixation method, radial load (N)	Crosswire	-	Parallel Wire	47.8	9.18	<0.001	4.33
		-	Single Wire	189.4	36.36	<0.001	17.14
	Parallel Wire	-	Single Wire	141.6	27.17	<0.001	12.81
Single wire fixation with shifted exit point, dorsal load (N)	D10	-	D5	7.94	2.093	0.243	0.987
		-	Single Wire	2.19	0.578	0.978	0.273
		-	P5	−4.6	−1.211	0.745	−0.571
		-	P10	−14.18	−3.738	0.005	−1.762
	D5	-	Single Wire	−5.75	−1.515	0.559	−0.714
		-	P5	−12.53	−3.305	0.016	−1.558
		-	P10	−22.12	−5.831	<0.001	−2.749
	Single Wire	-	P5	−6.79	−1.79	0.394	−0.844
		-	P10	−16.37	−4.316	<0.001	−2.034
	P5	-	P10	−9.58	−2.526	0.105	−1.191
Single wire fixation with shifted exit point, radial load (N)	D10	-	D5	0.0441	0.0119	1	0.00561
		-	Single Wire	9.2868	2.503	0.11	1.17993
		-	P5	15.6805	4.2263	0.001	1.99229
		-	P10	13.4185	3.6166	0.007	1.70489
	D5	-	Single Wire	9.2427	2.4911	0.113	1.17433
		-	P5	15.6364	4.2144	0.001	1.98668
		-	P10	13.3744	3.6047	0.007	1.69929
	Single Wire	-	P5	6.3937	1.7233	0.432	0.81235
		-	P10	4.1318	1.1136	0.798	0.52496
	P5	-	P10	−2.2619	−0.61	0.973	−0.28739
Single wire fixation with shifted exit point, Torsion 5° (Nm)	D10	-	D5	0.03055	2.443	0.125	1.151
		-	Single Wire	0.03406	2.724	0.068	1.284
		-	P5	0.00935	0.747	0.944	0.352
		-	P10	0.04015	3.211	0.021	1.513
	D5	-	Single Wire	0.00352	0.281	0.999	0.132
		-	P5	−0.0212	−1.695	0.448	−0.799
		-	P10	0.0096	0.768	0.938	0.362
	Single Wire	-	P5	−0.02472	−1.976	0.296	−0.932
		-	P10	0.00609	0.487	0.988	0.23
	P5	-	P10	0.03081	2.463	0.12	1.161
Single wire fixation with shifted exit point, Torsion 10° (Nm)	D10	-	D5	0.03689	3.032	0.033	1.429
		-	Single Wire	0.04135	3.4	0.013	1.603
		-	P5	0.04482	3.684	0.006	1.737
		-	P10	0.07615	6.26	<0.001	2.951
	D5	-	Single Wire	0.00447	0.367	0.996	0.173
		-	P5	0.00793	0.652	0.965	0.307
		-	P10	0.03927	3.228	0.02	1.522
	Single Wire	-	P5	0.00346	0.285	0.999	0.134
		-	P10	0.0348	2.861	0.049	1.348
	P5	-	P10	0.03134	2.576	0.094	1.214

* Cohen’s d: standardized difference between two means. If d < 0.2 the difference between means is negligible, d > 0.8 is considered a large effect size.

## Appendix E

**Table A5:** Results of the Kruskal–Wallis tests for the different fixation methods (Group I, II, and III) during the 5° and 10° torsion tests. The level of significance was set to 5% (α = 0.05).

Kruskal–Wallis				
	χ^2^	df	*p*	ε^2^
Torsion 5° (Nm)	15.6	2	<0.001	0.601
Torsion 10° (Nm)	17.7	2	<0.001	0.681

## Appendix F

**Table A6:** Results of the post hoc (Dwass–Steel–Critchlow–Fligner pairwise comparisons) tests for the different fixation methods (Group I, II, and III) during the 5° and 10° torsion tests. The level of significance was set to 5% (α = 0.05). Statistically significant (*p* *<* 0.05) results are highlighted with light gray.

Post Hoc Tests—Results					
				W	*p*
Torsion 5° (Nm)	Crosswire	-	Parallel Wire	3.06	0.078
	Crosswire	-	Single Wire	−3.43	0.04
	Parallel Wire	-	Single Wire	−5.06	0.001
Torsion 10° (Nm)	Crosswire	-	Parallel Wire	2.19	0.27
	Crosswire	-	Single Wire	−4.81	0.002
	Parallel Wire	-	Single Wire	−5.06	0.001

## Appendix G

**Table A7:** Descriptive statistics of the single wire group and subgroups (Group III, Group III/D10, D5, P5, P10). The table contains the means, the medians, the standard deviations, the variances, and the Shapiro–Wilk W and *p* values.

Descriptive Statistics					
	Fixation Method	Dorsal Load (N)	Radial Load (N)	Torsion 5° (Nm)	Torsion 10° (Nm)
Mean	D10	118	176	0.192	0.263
	D5	110	176	0.162	0.226
	Single wire	116	166	0.158	0.222
	P5	123	160	0.183	0.219
	P10	132	162	0.152	0.187
Median	D10	120	175	0.192	0.265
	D5	111	175	0.163	0.226
	Single wire	116	162	0.155	0.218
	P5	121	160	0.172	0.226
	P10	134	162	0.146	0.177
Standard deviation	D10	9.03	7.98	0.0152	0.0133
	D5	9.69	10.4	0.019	0.0199
	Single wire	5.15	9.45	0.0303	0.0272
	P5	3.19	6.28	0.0283	0.0294
	P10	10.6	3.15	0.0347	0.0339
Variance	D10	81.6	63.7	2.30 × 10^−4^	1.77 × 10^−4^
	D5	93.9	107	3.62 × 10^−4^	3.98 × 10^−4^
	Single wire	26.5	89.2	9.21 × 10^−4^	7.37 × 10^−4^
	P5	10.2	39.5	8.01 × 10^−4^	8.65 × 10^−4^
	P10	112	9.95	0.00121	0.00115
Shapiro–Wilk W	D10	0.912	0.945	0.886	0.929
	D5	0.956	0.956	0.962	0.963
	Single wire	0.933	0.781	0.974	0.96
	P5	0.858	0.924	0.844	0.927
	P10	0.836	0.907	0.97	0.98
Shapiro–Wilk *p*	D10	0.331	0.636	0.183	0.476
	D5	0.754	0.755	0.816	0.833
	Single wire	0.509	0.012	0.927	0.796
	P5	0.091	0.424	0.063	0.451
	P10	0.052	0.295	0.899	0.964
